# An In Situ Synchrotron Dilatometry and Atomistic Study of Martensite and Carbide Formation during Partitioning and Tempering

**DOI:** 10.3390/ma14143849

**Published:** 2021-07-09

**Authors:** Ernst Plesiutschnig, Mihaela Albu, David Canelo-Yubero, Vsevolod I. Razumovskiy, Andreas Stark, Norbert Schell, Gerald Kothleitner, Coline Beal, Christof Sommitsch, Ferdinand Hofer

**Affiliations:** 1 Institute of Materials Science, Joining and Forming, Graz University of Technology, Kopernikusgasse 24/I, 8010 Graz, Austria; caneloyubero@ujf.cas.cz (D.C.-Y.); coline.beal@tugraz.at (C.B.); christof.sommitsch@tugraz.at (C.S.); 2 Graz Centre for Electron Microscopy, Steyrergasse 17, 8010 Graz, Austria; mihaela.albu@felmi-zfe.at (M.A.); gerald.kothleitner@felmi-zfe.at (G.K.); ferdinand.hofer@tugraz.at (F.H.); 3 Nuclear Physics Institute ASCR, Řež, CZ-25068 Prague, Czech Republic; 4 Materials Center Leoben Forschungsgesellschaft GmbH, Roseggerstraße 12, 8700 Leoben, Austria; Vsevolod.Razumovskiy@mcl.at; 5 Institute of Materials Research, Helmholtz-Zentrum Geesthacht, Max-Planck-Str. 1, 21502 Geesthacht, Germany; andreas.stark@hereon.de (A.S.); norbert.schell@hereon.de (N.S.); 6 Institute of Electron Microscopy and Nanoanalysis, Graz University of Technology, Steyrergasse 17, 8010 Graz, Austria

**Keywords:** stainless steel, quenching and partitioning heat treatment, martensite, reconstructive ferrite, carbide formation, partitioning and tempering, high-resolution transmission electron microscopy, atomistic study, density functional theory, in-situ synchrotron study

## Abstract

Precipitation hardened and tempered martensitic-ferritic steels (TMFSs) are used in many areas of our daily lives as tools, components in power generation industries, or in the oil and gas (O&G) industry for creep and corrosion resistance. In addition to the metallurgical and forging processes, the unique properties of the materials in service are determined by the quality heat treatment (HT). By performing a quenching and partitioning HT during an in situ high energy synchrotron radiation experiment in a dilatometer, the evolution of retained austenite, martensite laths, dislocations, and carbides was characterized in detail. Atomic-scale studies on a specimen with the same HT subjected to a laser scanning confocal microscope show how dislocations facilitate cloud formation around carbides. These clouds have a discrete build-up, and thermodynamic calculations and density functional theory explain their stability.

## 1. Introduction

Over a century, the Fe-Cr-C system has been used to develop wear-, heat-, and corrosion-resistant martensitic hardenable steels for many industrial applications. Industry utilizes the martensitic transformation to achieve homogeneous properties such as tensile strength (often higher than 2.5 GPa) that are not possible with grain-refinement, cold working, or precipitation strengthening over a wide dimensional range. Cutlery/surgical instruments, turbine blades, or rotors are examples of light and heavy cross sections, respectively. Additions of alloying elements such as nickel, tungsten, or molybdenum to the Fe-Cr-C system improve hardenability and lead to increased high-temperature strength, in combination with vanadium and carbon, to form harder and more wear-resistant steels [1]. To develop such materials for the specific application, a martensitic stainless steel such as X20Cr13 (1.4021, AISI 420) is a good point of reference because of its wide applicability and availability despite being relatively simply alloyed. This is helpful to investigate heat treatment (HT)-dependent structure–property relationships.

An example is the study of austenite reversion for the transformation-induced plasticity (TRIP) effect [2,3], which contributes to the increase of ductility. The amount of retained austenite and carbides formed during this type of HT can be influenced by additions of Si [4]. The partitioning temperature to achieve this microstructure type is usually below 600 ${}^{{}^{\circ}}$C and is suitable for low temperature applications.

For applications at medium or high temperatures, it is important to obtain a stable microstructure. The tempering temperature should thus be selected at least 80–100 ${}^{{}^{\circ}}$C above the application temperature. As in a typical quenching and partitioning (Q&P) process, the austenite (γ) is split into deformed γ plus martensite ($\alpha^{\prime }$) in a first step, but combined with a diffusive γ-to-ferrite (α) phase transformation in a second step, leading to an increase of creep strength and ductility, i.e., an increase of component lifetime and increased efficiency (CO${}_{2}$ savings) [5,6]. For the purpose of application, high temperature strength and ductility are important to produce fail-safe components for operating temperatures up to 650 ${}^{{}^{\circ}}$C, therefore it is also important to avoid residual austenite, which is the reason for subsequent second tempering. This HT and microstructure without the second tempering will be studied using an industrial model alloy.

Quality HT combined with the selection of appropriate alloying elements requires an understanding of the application, microstructure with property-determining phases, solubility and mobility of alloying elements to describe the desired metastable states or pathways to equilibrium. This description of the nature steps is necessary to advance in physical models and computational tools. Robson and Bhadeshia [7,8] investigated the precipitation sequence in a 9Cr1Mo type steel, Schneider and Inden [9] did so for a 12%Cr steel, and Hou et al. [10] described the evolution for cementite in an early stage on a low alloyed 1C-1Cr containing steel. Based on the stage and chemical composition of carbide precipitation, it is possible to understand how much diffusion is required to precipitate carbides of one type, which allows conclusions to be drawn about the interaction between processes in the matrix and carbide formation.

Without claiming to provide a complete list for reference, allotropic phase transformations in steels are explained by a great amount of research. The description of steps and driving forces for the α or $\alpha^{\prime }$ formation from parent γ demonstrates the depth of understanding [11,12,13,14,15]. The martensite start (M${}_{s}$) temperature is quite similar for different variants of this steel, the main influence being the forcibly dissolved carbon content in the matrix [16,17,18]. However, a combination of phase transformations increases complexity and leads to rather less well-reported cases. Using a combination of quenching, partitioning, and annealing, we observe the influence of a non-diffusive $\gamma \to \alpha^{\prime }$-transformation on the subsequent diffusive γ→α-transformation, with the kinetics of the latter being enhanced by the first transformation at annealing temperature [19]. This combination of deformed γ and $\alpha^{\prime }$ at M${}_{s}$ temperature adds complexity (e.g., tracking the initially formed γ|$\alpha^{\prime }$ interface) compared with cooling from solution annealed γ below martensite finish (M${}_{f}$) temperature. We have previously studied such moving interfaces and recovery processes driven by enhanced diffusion of alloying elements [6,20].

This work focuses on the description of the metastable steps for the formation of thermodynamically stable phases after and during a Q&P HT. For high-resolution atomic-scale scanning transmission electron microscopy HR STEM studies, a sample was first subjected to the Q&P HT using a high temperature laser scanning confocal microscopy (LSCM, VL-2000DX, Yonekura, Lasertec Corp., Yokohama, Japan) [20] for extracting the smaller STEM lamella at the desired position. In situ synchrotron dilatometry was used for studies during Q&P HT. Both techniques coupled with density functional theory (DFT) computations allow the calculation of the formation energy of metastable states after quenching, partitioning, and tempering [21,22] to provide a picture of the physical processes that occur during and after the HT. The emphasis is on observations and first principle calculations of driving forces, but not on modelling the transformation or kinetics of carbide formation [19,23].

The main objective of this work is to characterize the microstructure of this stainless steel after Q&P and tempering to investigate the mechanism of stable carbide formation and the associated delayed diffusive γ→α transformation. Showing how metastable M${}_{3}$C carbides can precipitate and evolve by the diffusion of Fe, Cr, and C atoms, we see the formation of cloud-like regions and how dislocations play their role in this process. The evolution of carbide is important in describing the evolution of this class of steel, but carbide or cementite in particular can also be seen in a much broader context [24]. The material used for this study is an industrial X20Cr13 (AISI 420) stainless steel with the chemical composition and transformation temperatures given in Table 1.

## 2. Materials and Methods

### 2.1. Sample Preparation Details for the STEM Imaging

**Figure 1:**
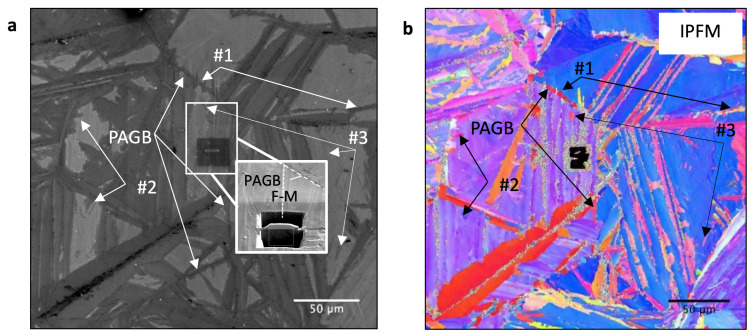
(**a**,**b**) Scanning electron microscopy (SEM) and IPFM images after the same Q&P heat treatment (HT) (as in Figure 2) performed with a high temperature laser scanning microscope [20]. The magnification in (**a**) indicates the removal position of the lamella used here for further more in-depth investigations.

The lamella from the tempered martensitic steel in Figure 1 was prepared by focused ion beam (FIB) milling and is approximately 8 × 4 µm (large × width) and 150 nm thick. To bring the lamella to a thickness of about 50 nm and remove the formed amorphous layer on the surface, two subsequent low energy Argon ion milling post–treatments at 900 eV (56 µA at ±10° and each side 15 min) were applied using the NanoMill device. The 20–40 nm thick regions at the top of the specimen were used for atomic resolution imaging and analytical investigations.

### 2.2. TEM Technique and Image Analysis

For imaging and analytics in scanning mode at atomic level, an advanced hardware and manual optimization is required. A probe aberration-corrected microscope FEI Titan3 2 G 60–300 kV (Thermo Fisher Scientific Inc., Breda, The Netherlands), equipped with X-FEG Schottky field-emission electron source, Super-X detector (Chemi-STEM technology, Thermo Fisher Scientific Inc., Breda, The Netherlands) consisting of four separate silicon drift detectors (0.7 sr collection angle) [28], and dual electron energy loss spectroscopy (EELS)-Gatan imaging filter Quantum (GIF, Gatan Inc., Pleasanton, CA, USA) [29], was used. The analysed tempered martensitic steel sample possesses its own magnetic field (consists of >87 wt% Fe) and required, for the atomic resolution, the optimization of the Cs-corrector element. The optimization is not straight forward because many magnetic domains with different orientations deflect the beam and influence the astigmatism correction. Nevertheless, an aberration-free zone of 26 mrad at 300 kV with a 50 µm condenser aperture was reached. The beam convergence was set to 19.6 mrad. High angular annular dark field (HAADF) and annular dark field (ADF) detectors were used to acquire the high resolution STEM (HR STEM) images.

### 2.3. In Situ Synchrotron Experiments

**Figure 2:**
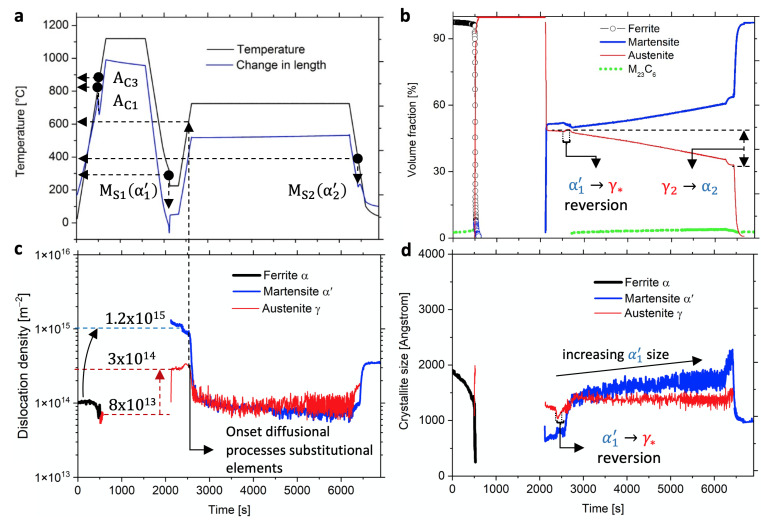
The Q&P heat treatment (HT) applied with the results from the in situ synchrotron radiation experiment combined with dilatometry show the volume fractions of different phases $\gamma ,\alpha ,\alpha^{\prime }$, and M${}_{23}$C${}_{6}$; the evolution of the internal dislocation density; and the crystallite size in (**a**–**d**).

In situ high energy X-ray diffraction (HEXRD) experiments were performed in transmission mode at the P07-HEMS beamline of Petra III, Deutsches Elektronen-Synchrotron (DESY), Hamburg [30]. A sketch of the setup can be found elsewhere [31]. A modified Bähr 805A/D dilatomer (TAInstruments, New Castle, DE, USA) [32], with two viewports and kapton windows for the incident and diffracted beams, was used for the thermal treatments with heating/cooling rates of 100 Kmin${}^{-1}$. A sample of 5 mm diameter and 10 mm length was tested during the experiments. The incident beam was set to 0.7 × 0.7 mm${}^{2}$ (H × V) and placed just below the thermocouple of type K. A sample-detector distance of 1493 mm and beam energy of 100 KeV (λ = 0.124 Å) allowed to collect the Debye–Scherrer rings in a 2D Perkin–Elmer detector with an array of 2048 × 2048 and pixel size of 200 × 200 µm. Different acquisition times were used to record the 2D images: Figure 2a 1s from A${}_{c1}$ (beginning of the α→γ transformation) up to the austenitization temperature (A${}_{\gamma }$), during subsequent cooling from A${}_{\gamma }$ up to the beginning of the tempering (T${}_{t}$), and during the final cooling down; Figure 2b 5 s during the initial heating, and both holding temperatures A${}_{\gamma }$ and T${}_{t}$. The collected 2D images were integrated into sectors using the Input4MAUD software (Version 2.8, University of Göttingen, Germany) [33] and Rietveld refinements were performed with MAUD software (Version 2.55, University of Trento, Italy) [34]. Goodness of the refinements was assessed with the weighted R${}_{wp}$ and R${}_{wnb}$ (no background) factors, which typically ranged from 10% to 20%. The dislocation densities were calculated using the Williamson–Smallman relationship (see Equation (1)), which takes into account the average of the internal dislocation density. The parameters *p* and *F* were set to 1 and k to 14.4 for the bcc/bct and 16.1 for the fcc lattices.

(1) $$\rho =\sqrt{\frac{3pk}{F}}\frac{e_{RMS}}{\vec{b}D_{eff}}$$

Equation (1) shows the William–Smallman relation [35] for the calculation of the internal dislocation density. ρ is the number of dislocations on the crystallite face of the block structure, *k* is defined by the lattice structure, *F* accounts for the energy interaction of dislocations, and $e_{RMS}$ is the microstrain provided by the Rietveld refinements together with the Burgers vector $\vec{b}$ and the crystallite size $D_{eff}$.

### 2.4. DFT Calculation Method

Spin polarized DFT calculations of the total energy have been performed using the projector-augmented-wave (PAW) method [36,37] as implemented in the Vienna ab initio simulation package (VASP) [38,39]. We have used the generalized gradient approximation (GGA) to the exchange correlation potential PBE [40,41]. The VASP-PAW calculations were performed using a plane-wave cut-off energy of 400 eV. The convergence criteria were chosen to be $10^{-5}$ eV for the total energy and 9 × $10^{-3}$ eV/A for the forces. Ionic relaxations were included in all calculations. The integration over the Brillouin zone was done using 4 × 4 × 4 Monkhorst-Pack scheme [42] for the 4 × 4 × 4 [conventional bcc cell] supercell and 12 × 12 × 12 for the Cr${}_{3}$C in D0${}_{11}$ (Fe${}_{3}$C-type) carbide structure with 16 atoms per cell. All calculations were converged to provide computational accuracy within 0.003 eV [25,27]. The configurational entropy contribution to the free energy at elevated temperatures was evaluated using an analytical expression for disordered alloys $S=\sum_{i}x_{i}lnx_{i}$, where $x_{i}$ is the atomic fraction of the *i*th species in the alloy. The phonon contribution to the free energy was taken into account in the framework of the Debye–Grüneisen model [43].

### 2.5. DFT Microstructure Setup

Bcc Cr-Fe-C disordered alloys (see Table 1) Fe${}_{73}$Cr${}_{27}$C${}_{0}$ (Matrix), Fe${}_{31}$Cr${}_{58}$C${}_{11}$ (Cloud 1), and Fe${}_{10}$Cr${}_{65}$C${}_{25}$ (Cloud 1) were modelled based on a 4 × 4 × 4 (conventional bcc cell) 128-substitutional site supercell (SC). Carbon atoms are positioned at the interstitial octahedral sublattice in the amount proportional to the atomic composition of steel. Atomic disorder on both metal and C-sublattices was controlled via pair correlation functions. Only SCs with as close as possible to zero (complete disorder) pair correlation functions at the first 8 coordinations and 15 clouds were selected to represent alloys listed above. Atomic disorder on Me- and C- sublattices was treated independently of each other. All structures were rendered using the visualization for electronic structural analysis (VESTA) 3D [44] software (Version 3, Copyright (C) 2006-2017, Koichi Momma and Fujio Izumi, Ibaraki, Japan).

## 3. Results and Discussion

Figure 2a–d show the results from the in situ synchrotron radiation experiment. Figure 2a shows the experimental Q&P HT along with the dilatometry response; Figure 2b shows the volume fraction of the carbide phase with the matrix phases austenite (γ) and ferrite (α) (or martensite ($\alpha^{\prime }$) morphology); Figure 2c the dislocation densities; and Figure 2d shows the average size of the coherent scattering domains (referred to here as crystallite).

**Figure A1:**
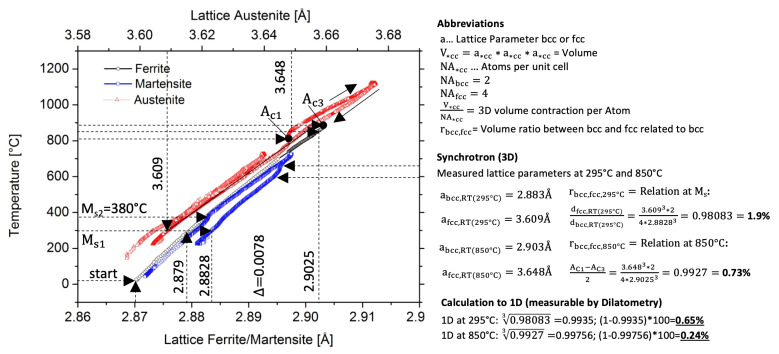
Shows the evolution of the lattice parameters for fcc and bcc (bct) during the in situ synchrotron experiment with the calculated volumetric expansion and relaxation in 3D and 1D for comparison.

**Heat treatment and matrix formation:** Starting from a polycrystalline tempered martensitic microstructure, we follow the time starting from 0. A${}_{c1}$ indicates the first presence of γ until complete austenitization at A${}_{c3}$. With increasing temperature, the carbides coarsen in a first step by Ostwald ripening (LSW theory) and dissolve in γ in the second step. Solution annealing at 1050 ${}^{{}^{\circ}}$C dissolves all carbides; subsequent cooling to 265 ${}^{{}^{\circ}}$C (between M${}_{s1}$ and M${}_{f1}$ at 2200 s time) partitions the γ matrix into $\alpha_{1}^{\prime }$ and mechanically stabilized deformed γ (see Figure 2b). The formation of $\alpha_{1}^{\prime }$ increases the dislocation density (ρ) in $\alpha_{1}^{\prime }$ to 1.2 × $10^{15}$ [m${}^{-2}$] and to 3 × $10^{14}$ [m${}^{-2}$] in γ owing to the lattice expansion of $\alpha_{1}^{\prime }$ (see Figure 2c). This expansion is responsible for the mechanical stabilization of γ and the amount of formed $\alpha_{1}^{\prime }$ at a chosen partitioning temperature. From the perspective of lattice parameter evolution (Figure A1), the formation of γ reduces the matrix volume by only 0.73% at 850 ${}^{{}^{\circ}}$C (between A${}_{c1}$ and A${}_{c3}$), but then expands to $\alpha_{1}^{\prime }$ by 1.9% at M${}_{s1}$ ($\gamma \to \alpha_{1}^{\prime }$). This volume expansion releases the free energy acquired by γ during cooling from A${}_{r1}$ to M${}_{s1}$ and is measured indirectly by dilatometry and synchrotron for the different linear thermal expansion coefficients of the bcc and fcc lattices, i.e., 1.3 × $10^{-5}$K${}^{-1}$ and 2.2 × $10^{-5}$K${}^{-1}$, respectively. M${}_{s}$ is reached when the chemical driving force is sufficient to cause dislocation assemblies to shear through the parent γ-lattice [12,45]. This movement of dislocations at ultrasonic velocity introduces voids, interfaces, strain, and latent heat due to lattice friction. Upon reheating this split γ and $\alpha_{1}^{\prime }$ matrix, the microstructure evolves towards the tempering temperature, austenite reversion, and recovery, and the reconstructive γ→α transformation follows after sufficient mobility of Fe and Cr. The subsequent tempering at 725 ${}^{{}^{\circ}}$C with a partitioned volume fraction (vol.%) of $50_{\gamma }:50_{\alpha_{1}^{\prime }}$ ends with $38_{\gamma }:50_{\alpha_{1}^{\prime }}+12_{\alpha }$ before the final cooling step. During this last step, the remaining $38_{\gamma }$ vol.% transforms into $\alpha_{2}^{\prime }$ at M${}_{s2}$ (400 ${}^{{}^{\circ}}$C). This increase in transformation temperature (ΔT = M${}_{s2}$− M${}_{s1}$ = 100 ${}^{{}^{\circ}}$C) indicates a decrease in chemical driving force required for $\alpha_{2}^{\prime }$ formation, a decrease in the equilibrium yield stress of the parent fcc lattice, and a lower C concentration within the deformed and partitioned γ.

**Austenite reversion:** Upon heating from the partitioning temperature toward the tempering temperature (from 265 ${}^{{}^{\circ}}$C to 725 ${}^{{}^{\circ}}$C), the lattice parameter (Figure A1) of $\alpha_{1}^{\prime }$ decreases by 0.6% at 330 ${}^{{}^{\circ}}$C, indicating the release of C from interstitial positions toward dislocations and interfaces; at about 500 ${}^{{}^{\circ}}$C, the austenite reversion affects the γ crystallite size, as observed in Figure 2d and evidenced by a decrease. Reversion is reported to take place at the $\alpha_{1}^{\prime }$|γ interfaces where $\alpha_{1}^{\prime }$ reverts into $\gamma_{*}$ nano-sized crystallites, caused by local equilibrium through C segregation at high local concentrations [2].

**Recovery** is important to reduce the plastic strain resulting from the transformation, release mechanical stabilization of γ, and increase the low ductility of fresh formed $\alpha_{1}^{\prime }$ blocks. If the $\gamma \to \alpha_{1}^{\prime }$ transformation proceeds with further undercooling, the already indicated 1.9% local volume increase may burst these untempered $\alpha_{1}^{\prime }$ blocks. Increasing diffusivity at 600 ${}^{{}^{\circ}}$C accelerates recovery of $\alpha_{1}^{\prime }$ and shows the limit of this morphology for use at higher temperatures. We observed a higher onset of this temperature (680 ${}^{{}^{\circ}}$C) for a steel with higher stability [6] after performing the similar experiment.

**Ferrite (α) and carbide formation** starts at a tempering temperature of 725 ${}^{{}^{\circ}}$C, when sufficient energy is available for the diffusion of substitutional elements such as Cr at dislocations and interfaces in parent γ and in $\alpha_{1}^{\prime }$. Carbide formation is a prerequisite for the growth of α so that the γ→α-reaction can proceed stress-free [46]. We observe M${}_{3}$C and M${}_{23}$C${}_{6}$ carbides during the tempering, with the former having a low vol.% content combined with a small crystallite size, which hinders their quantitative analysis (Figure 2). Their high Cr content has negative corrosive effects (sensitization) [47,48], but is highly desired for decreasing coarsening to prevent recovery [49]. Because of their importance, we devote the following sections to answering the question of their formation.

We applied the similar Q&P HT in situ to a specimen in a laser scanning confocal microscopy (LSCM) experiment [20]. This sample was subsequently analysed by scanning electron microscopy (SEM) to show the obtained ferrite morphologies in Figure 1. With classified by Dubé’s sceme [15], it is possible to distinguish between $\alpha_{1}^{\prime }$ during partitioning, $\alpha_{2}^{\prime }$ transformed from residual γ, and two types of reconstructive ferrite (α) morphologies formed at different nucleation sites. One type grows acicular along the $\alpha_{1}^{\prime }$ block boundaries #1, #2 (allotriomorphic), and another #3 inside γ (idiomorphic) incorporated in the later transformed $\alpha_{2}^{\prime }$ block. For HR STEM investigations, we cut the lamella from the interfaces.

**Figure 3:**
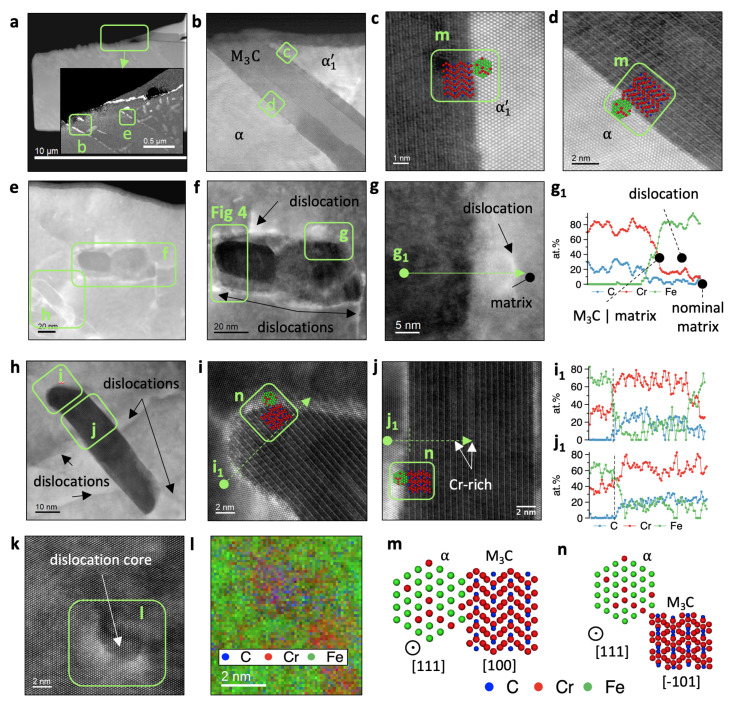
HR STEM investigations for the focused ion beam (FIB) lamella (see Figure 1a). (**a**) An overview of the FIB lamella zooming into the area of interest-magnified and indicated in (**b**,**e**) in the Cr-EFTEM map. (**b**) A high resolution STEM (HR STEM) image of the inter-phase M${}_{3}$C coherent to the reconstructive grown α matrix in (**c**) and the tempered $\alpha_{1}^{\prime }$ at the other side in (**d**,**e**) shows two intra-phase M${}_{3}$C with attached dislocations (indicated with dark arrows) magnified in (**f**,**h**) indicated with dark arrows. Further indicated for both carbides are the recorded electron energy loss spectroscopy (EELS) chemical line profiles (**g1**,**i1**,**j1**) for the elements C, Fe, and Cr. The magnified M${}_{3}$C in (**f**) is surrounded by two clouds, while the M${}_{3}$C in (**i**) is surrounded only by a thin cloud. The HAADF in (**k**) shows a dislocation core inside the tempered $\alpha_{1}^{\prime }$ block analysed with the EELS, and the composed RGB image in (**l**) contains elemental maps showing an enrichment of Cr and C inside the dislocation core. (**m**,**n**) The orientation of M${}_{3}$C relative to the matrix.

In Figure 3a–l, we present HR STEM investigations depicting the microstructure of the FIB lamella using different detectors up to atomic resolution. Figure 3m,n illustrate the arrangement of atoms between α (tempered $\alpha_{1}^{\prime }$) and M${}_{3}$C, while the images with the indexes Figure 3g1,i1,j1) are quantified electron energy loss (EELS) line-scans and correspond to indicated locations of the parent pictures. The overview in Figure 3a shows the FIB lamella with the inset being an energy-filtered TEM image of Cr distribution acquired in the region marked with a rectangle. Two types of carbides were observed in the tempered $\alpha_{1}^{\prime }$ blocks with distinguishable carbide morphologies inside. One type of carbide grows acicular along the $\alpha_{1}^{\prime }$ block boundaries (interface carbide) and the other idiomorph inside the $\alpha_{1}^{\prime }$ block (intra-phase carbide)—similar to the α-morphologies indicated in the Electron Backscatter Diffraction (EBSD) measurements (see Figure 1). Figure 3b emphasizes the interface carbide separating $\alpha_{1}^{\prime }$ from the reconstructive grown α (Figure 3d). This carbide initially grows acicular along the partitioned $\gamma |\alpha_{1}^{\prime }$ interface and enables the γ→α transformation during the tempering through C depletion of the surrounding γ. Both sides share the similar orientation relationship with the M${}_{3}$C carbide ${\left[111\right]}_{\mathrm{bcc}}\left|\right|\left[100\right]{}_{M_{3}C}$ despite their different times of evolution. Dislocations are nucleation sites for intra-phase M${}_{3}$C carbides (see Figure 3k). We measure concentrations between 20 and 36 at% of Cr and 5 and 10 at% of C inside the dislocation cores attached to the M${}_{3}$C carbides (see Figure 3f,h), thus they supply their growth with Cr and C atoms through pipe diffusion. In Figure 3h, we observe the intra-phase carbide surrounded by dislocations and the tempered $\alpha_{1}^{\prime }$ block, further magnified in Figure 3i–j. Both positions demonstrate an ordered arrangement of alternating Cr-Fe and C atoms within the orthorhombic crystal structure. The carbide core is surrounded by a thin cloud of 3–4 atomic layers with a lower Cr concentration of about 1 at% than the core on both sides coherent towards the matrix. The bright diffraction contrast is probably caused by coherency stresses (misfit) or by the strain induced in the matrix due to disordered Cr enrichment and the induced vacancies. The orientation of the carbide relative to the matrix ${\left[111\right]}_{\mathrm{matrix}}\left|\right|{\left[-101\right]}_{M_{3}C}$ is different compared with the inter-phase M${}_{3}$C above (Figure 3f,g). Overlapping EELS element profiles for Fe, Cr, and C in Figure 3i1,j1 indicate the M${}_{3}$C stoichiometry. Figure 3j further shows local variations of Fe and Cr content inside the carbide. The next intra-phase carbide in Figure 3e–g1 deserves our attention because of two surrounding clouds that help the carbide to follow an interesting path towards Cr and C enrichment. Identical to the previous ones, dislocations provide C and Cr atoms to the carbide. The carbide core has a chemical composition of 25% C and 75% Cr and an orthorhombic crystal structure with a lattice parameter 3% smaller than the clouds. The clouds’ lattice parameters are similar to the bcc matrix, while the M${}_{3}$C core is oriented ${\left[111\right]}_{\mathrm{matrix}}\left|\right|{\left[-103\right]}_{M_{3}C}$.

**Figure 4:**
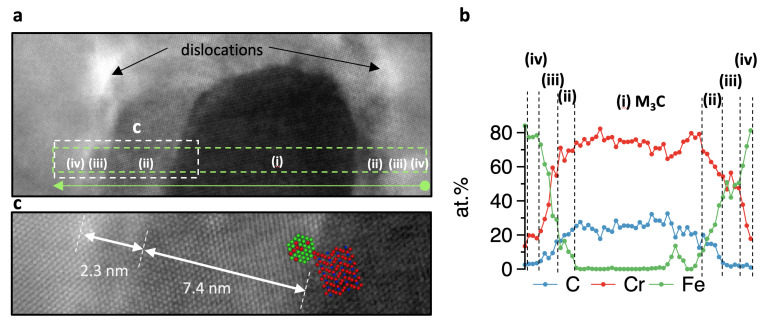
(**a**) The HR STEM magnification of Figure 3f with dislocations, (i) the carbide core, two clouds (ii), (iii) with different chemical compositions, and the Cr enriched bcc matrix (iv). The small highlighted area is shown magnified in (**c**), while the arrow with EELS at the bottom of (**a**) indicates the path of the plotted chemical line profile with EELS starting from right to left, shown in (**b**).

Following the HR STEM image in Figure 3f, magnified in Figure 4a,c with the chemical line profile in Figure 4b, we distinguish four zones with different compositions/crystal structures: (i) M${}_{3}$C carbide (Core) in DO${}_{11}$ orthorhombic crystal structure with the lattice constants listed in Table A1 and the following chemical composition: C = 24.4±3.4 at%, Cr = 75.6±3.8 at%, and Fe = 0; (ii) Cloud 1 of 7.4 nm in length covering the carbide: bcc random solid solution with C = 24±3.6 at%, Cr = 65.7±4.1 at%, and Fe = 10.3±2.6 at%; (iii) Cloud 2 of 2.3 nm in length surrounding the carbide: bcc random solid solution with C = 13±6.6 at%, Cr = 58±1.7 at%, and Fe = 29±5.2 at%; and (iv) bcc matrix (Matrix), which is primarily represented by a disordered Fe${}_{73}$Cr${}_{27}$ solid solution. The crystal structure for both clouds is similar to the bcc matrix. On the STEM image in Figure 4c, we observed a coherency of the latter three zones with a lattice constant of 2.8705 Å (value calculated from the synchrotron 2D images at room temperature).

**Figure 5:**
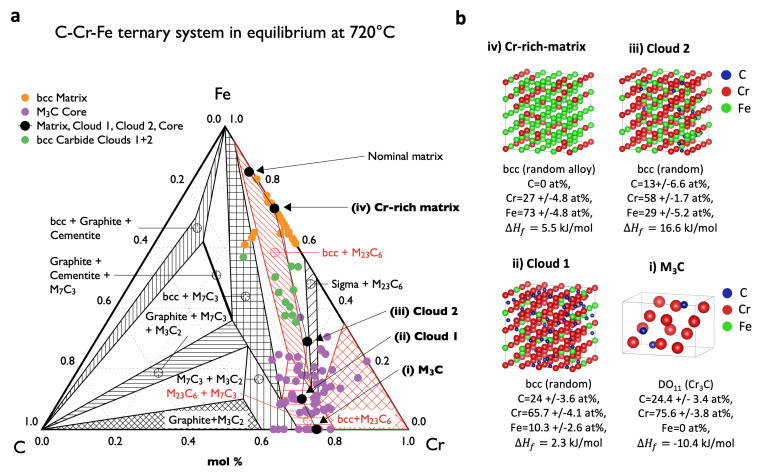
(**a**) The ternary Fe-Cr-C system at 720 ${}^{{}^{\circ}}$C with equilibrium data taken from FactSage [50]. The dots indicate data from the chemical profile in Figure 4b, for the M${}_{3}$C core and bcc carbide Clouds 1 and 2 used for the DFT calculations in (**b**).

This distinction between nominal matrix, Cr-enriched matrix, and Cloud 1 and 2 is shown inside the isothermal ternary C-Cr-Fe system in Figure 5a. Plotting the recorded EELS line profile of Figure 4b into Figure 5a confirms the visual assumption that a viewer of Figure 4a,c has, that the clouds have discrete chemical analyses. Based on these findings, we performed a series of DFT calculations of the structures (illustrated in Figure 5b to describe changes in the phase stability during precipitation of M${}_{3}$C.

Enthalpies of formation $\Delta H_{f}$ for the Core, Cloud 1, Cloud 2, and Matrix are calculated with the experimentally determined lattice parameters in Table 1 using 0K equilibrium crystal structures of ferromagnetic bcc Fe (a = 2.83 Å), nonmagnetic bcc Cr (a = 2.85 Å), and C in diamond structure (a = 3.56 Å). The results for the Core of the precipitate and the Matrix represented by Fe${}_{73}$Cr${}_{27}$ random alloys are in very close agreement (within a few kJ·mol${}^{-1}$) with the available literature data [25,26,27]. Fe${}_{73}$Cr${}_{27}$ random alloy (Matrix) has a positive $\Delta H_{f}$ and is prone to spinodal decomposition at low temperatures [27]. This remarkable finding suggests that the precipitation of coherent M${}_{3}$C (and presumably other carbide phases) in α can be determined by the driving forces from the difference between a supersaturated matrix with C in solid solution (Cloud 1 and Cloud 2 in Figure 5a) and the carbide phase, rather than from the difference between the ground state of the matrix with C in solid solution (Matrix in Figure 5a) and the precipitating carbide phase (M${}_{3}$C in Figure 5a).

It is further noticeable that an increase of the C content beyond the composition of Fe${}_{10}$Cr${}_{65}$C${}_{25}$ (we have calculated an additional alloy with 31 at% C) leads to a structural destabilization of the bcc phase. The obtained crystal structure has a very close resemblance to an amorphous state and possesses a very high $\Delta H_{f}$ (above 3000 kJ·mol${}^{-1}$). Regions with 20 and 25 at% C (Cloud 1) already exhibit similar strong structural relaxations (though not as strong as in the case of 31 at% C steel), indicating that any increase of the C content in the bcc phase of Cloud 1 would lead to a structural destabilization of this phase (with very high $\Delta H_{f}$) in favour of the DO${}_{11}$ phase.

## 4. Summary and Conclusions

The decay of austenite and the formation of carbide precipitates, as well as the physical and microstructural properties of an industrial X20Cr13 stainless steel during quenching, partitioning, and tempering, were studied. As is well known, conditions during air cooling from austenitization to room temperature result in the formation of martensite.

- When cooling from the austenitizing temperature, the substitutional dissolved portion of Cr in the matrix coupled with the high affinity of Cr for the element C retains C in the matrix and prevents the rapid precipitation of stable carbide. The lattice parameter of the parent phase (austenite) shrinks faster than the thermodynamically stable phase (ferrite). Their measurable difference plus the compressive strain on the local yield stress is represented by the martensitic expansion at M${}_{s}$ temperature.
- The martensitic expansion dissipates heat and separates the austenitic parent matrix into deformed retained austenite and martensite. Heterogeneous nucleation sites (voids, dislocations, and interfaces) are created during partitioning for the subsequent tempering step.
- Tempering above 600 ${}^{{}^{\circ}}$C leads to immediate recovery, thermally activated coarsening of martensite crystal laths (150 nm to 190 nm), carbide precipitation, and subsequent ferrite formation. The ferrite morphologies formed depend on the amount of locally forcibly dissolved C in the austenite and occur spontanously after local depletion by, for example, precipitation.
- Formation of metastable M${}_{3}$C carbide: Carbon segregates into dislocations and interfaces after partitioning. Cottrell atmospheres form in dislocations, and discrete enrichment with the element Cr occurs. The step-wise enrichment through the diffusion of Cr to discrete bcc random solid solutions such as Fe${}_{10}$Cr${}_{65}$C${}_{25}$, here referred to as clouds, takes time and is necessary for the nearly Fe-free Cr${}_{75}$C${}_{25}$ carbide to form.
- The determination of the driving force with density functional theory confirms that the energy state of the supersaturated bcc solid solution (cloud) is energetically favorable ($\Delta H_{f}$ = 3.2 kJ·mol${}^{-1}$) compared with the ground state of the matrix, leading to its formation. Thus, the coherent carbide (perhaps some other carbides too) is formed from the driving forces between the cloud and the carbide.
- Interphase and intraphase M${}_{3}$C carbides utilize nucleation and growth or spinodal decomposition for precipitation. Carbide also precipitates in deformed retained austenite during tempering, which explains the higher measured M${}_{s2}$ temperature on cooling from the tempering temperature of retained austenite.
- Cr-depleted zones are present prior to the existence of precipitates and may affect the corrosion resistance of the alloy by sensitization.
- In situ synchrotron studies provide an immediate estimate of the maximum operating temperature from the decrease in dislocation density between 580 and 600 ${}^{{}^{\circ}}$C. While this temperature increases for more heat-resistant steels, this experiment could save valuable time and expensive preliminary tests such as creep tests for new alloy developments.

## Figures and Tables

**Table 1:** Experimental lattice parameters a, b, and c (current work ((transmission electron microscopy (TEM) data; synchrotron data)) in Å and enthalpies of formation $\Delta H_{f}$ in kJ·mol${}^{-1}$ of bcc Fe${}_{73}$Cr${}_{27}$C${}_{0}$ (Matrix), bcc Fe${}_{31}$Cr${}_{58}$C${}_{11}$ (Cloud 2), and bcc Fe${}_{10}$Cr${}_{65}$C${}_{25}$ (Cloud 1) steels and DO${}_{11}$ Cr${}_{3}$C core. The obtained results for Fe${}_{73}$Cr${}_{27}$C${}_{0}$ and Cr${}_{3}$C are compared to theoretical literature data from [25,26].

	Composition			Lattice			$\Delta H_{f}$	$\Delta G_{1000K}$
	[at%]			Parameters			[kJmol${}^{-1}$]	[kJmol${}^{-1}$]
**#**	**Fe**	**Cr**	**C**	**a**	**b**	**c**		
Nominal	84	14	1	-	-	-	-	-
Matrix	73±4.8	14±4.8	0	2.87	2.87	2.87	5.5	−2
Cloud 2	29±5.2	58±1.7	13±6.6	2.87	2.87	2.87	16.6	0.8
Cloud 1	65.7±4.1	10.2±2.6	24±3.6	2.87	2.87	2.87	2.3	−6.2
M${}_{3}$C core	0	75.6±3.8	24.4±3.4	5.09	6.74	4.52	−10.4	-
M${}_{3}$C core	0	75.6±3.8	24.4±3.4	5.19	6.74	4.52	−10.4	-
(theory)							−7.3 [25]	
Matrix	-	-	-	2.84 [26]	2.84	2.84	−10.4	-
(theory)	-	-	-	2.83 [25]	2.83	2.83	395.6 [27]	-

## Data Availability

For details on the calculations of short range order (SRO) parameters (Appendix A), please contact V.I. Razumovskiy.

## Appendix A

**Table A1:** Temperature cycle with microstructure results after the conducted Q&P heat treatment for the X20Cr13 stainless steel.

#	Time/	Micro-	Temperature	$\rho \times 10^{14}$	Lattice
	Gradient	Structure	[${}^{{}^{\circ}}$C]	[m${}^{-2}$]	Parameter [Å]
Nominal	84	14	1	-	-
A${}_{c1}$	1.66 ${}^{{}^{\circ}}$C/s	$\alpha_{0}^{\prime }$	825	0.8	2.895, 3.650
A${}_{c3}$	1.66 ${}^{{}^{\circ}}$C/s	$\alpha_{0}^{\prime }$	875	50, 0.7	2.905, 3.660
T${}_{p}$	200 s	$\alpha_{1}^{\prime },\gamma$	290	18, 0.7	2.885, 3.605
	1.66 ${}^{{}^{\circ}}$C/s	$\alpha_{1}^{\prime },\gamma$	265	12, 3.0	(2.877) 2.885, 3.606
	1.66 ${}^{{}^{\circ}}$C/s	$\alpha_{1}^{\prime },\gamma$	265–400	11.5–9.5, 3.0–3.2	
	1.66 ${}^{{}^{\circ}}$C/s	$\alpha_{1}^{\prime },\gamma$	400–550	9.5–9.0, 3.2	
	1.66 ${}^{{}^{\circ}}$C/s	$\alpha_{1}^{\prime },\gamma$	550–610	9.0–8.0, 3.2–2.8	
	1.66 ${}^{{}^{\circ}}$C/s	$\alpha_{1}^{\prime },\gamma$	610–725	8.0–1.6, 2.8–1.2	
	1 h	$\alpha_{1}^{\prime },\gamma$	725	1.6–0.75, 1.2–0.7	2.898
	1.66 ${}^{{}^{\circ}}$C/s	$\alpha_{1}^{\prime },\gamma$	725–400	0.75–1.0, 0.7–2.0	
	1.66 ${}^{{}^{\circ}}$C/s	$\alpha_{1}^{\prime },\gamma ,\alpha_{2}^{\prime }$	400–220	1.0–3.0, 2.0–1.0	
	1.66 ${}^{{}^{\circ}}$C/s	$\alpha_{1}^{\prime },\gamma ,\alpha_{2}^{\prime }$	220–50	3.0–3.25, 10–200	

**Table A2:** CF for Matrix.

		Fe${}_{73}$Cr${}_{27}$C${}_{0}$ (Matrix)		<ss>	Alpha
		Alloy Components: Fe Cr			
<ss>	1	(−0.5000 −0.5000 −0.5000)	=	−0.203125	−0.002765
<ss>	2	(−1.0000 −0.0000 −0.0000)	=	−0.208333	−0.003789
<ss>	3	(−1.0000 −1.0000 −0.0000)	=	−0.203125	−0.002765
<ss>	4	(−1.5000 −0.5000 −0.5000)	=	−0.205729	0.000512
<ss>	5	(−1.0000 −1.0000 −1.0000)	=	−0.203125	−0.002765
<ss>	6	(−2.0000 0.0000 0.0000)	=	−0.21875	0.016897
<ss>	7	(−1.5000 −1.5000 −0.5000)	=	−0.203125	−0.002765
<ss>	8	(−2.0000 −1.0000 0.0000)	=	−0.166667	−0.048643

**Table A3:** CF for Cloud 1.

		Fe${}_{16}$Cr${}_{53}$C${}_{31}$ (Cloud 1)		<ss>	Alpha
		Alloy Components: Fe Cr			
<ss>	1	(−0.5000 −0.5000 −0.5000)	=	−0.296875	−0.003135
<ss>	2	(−1.0000 −0.0000 −0.0000)	=	−0.302083	0.004296
<ss>	3	(−1.0000 −1.0000 −0.0000)	=	−0.296875	−0.003135
<ss>	4	(−1.5000 −0.5000 −0.5000)	=	−0.299479	0.000581
<ss>	5	(−1.0000 −1.0000 −1.0000)	=	−0.296875	−0.003135
<ss>	6	(−2.0000 0.0000 0.0000)	=	−0.302083	0.004296
<ss>	7	(−1.5000 −1.5000 −0.5000)	=	−0.302083	0.004296
<ss>	8	(−2.0000 −1.0000 0.0000)	=	−0.276042	−0.032857
		Alloy Components: C Em (empty)		<ss>	Alpha
<ss>	1	(−0.5000 −0.5000 −0.5000)	=	−0.15625	−0.000515
<ss>	2	(−1.0000 −0.0000 −0.0000)	=	−0.159722	0.003603
<ss>	3	(−1.0000 −1.0000 −0.0000)	=	−0.15625	−0.000515
<ss>	4	(−1.5000 −0.5000 −0.5000)	=	−0.15625	−0.000515
<ss>	5	(−1.0000 −1.0000 −1.0000)	=	−0.15625	−0.000515
<ss>	6	(−2.0000 0.0000 0.0000)	=	−0.161458	0.005661
<ss>	7	(−1.5000 −1.5000 −0.5000)	=	−0.165365	0.010293
<ss>	8	(−2.0000 −1.0000 0.0000)	=	−0.166667	0.011837

**Table A4:** CF for Cloud 2.

		Fe${}_{31}$Cr${}_{58}$C${}_{11}$ (Cloud 2)		<ss>	Alpha
		Alloy Components: Fe Cr [B]			
<ss>	1	(−0.5000 −0.5000 −0.5000)	=	−0.085938	−0.00241
<ss>	2	(−1.0000 −0.0000 −0.0000)	=	−0.083333	−0.005266
<ss>	3	(−1.0000 −1.0000 −0.0000)	=	−0.088542	0.000446
<ss>	4	(−1.5000 −0.5000 −0.5000)	=	−0.085938	−0.00241
<ss>	5	(−1.0000 −1.0000 −1.0000)	=	−0.085938	−0.00241
<ss>	6	(−2.0000 0.0000 0.0000)	=	−0.09375	0.006158
<ss>	7	(−1.5000 −1.5000 −0.5000)	=	−0.078125	−0.010977
<ss>	8	(−2.0000 −1.0000 0.0000)	=	−0.083333	−0.005266
		Alloy Components: C Em (empty)		<ss>	Alpha
<ss>	1	(−0.5000 −0.5000 −0.5000)	=	−0.713542	0.00565
<ss>	2	(−1.0000 −0.0000 −0.0000)	=	−0.715278	0.011676
<ss>	3	(−1.0000 −1.0000 −0.0000)	=	−0.710938	−0.00339
<ss>	4	(−1.5000 −0.5000 −0.5000)	=	−0.711806	−0.000377
<ss>	5	(−1.0000 −1.0000 −1.0000)	=	−0.710938	−0.00339
<ss>	6	(−2.0000 0.0000 0.0000)	=	−0.713542	0.00565
<ss>	7	(−1.5000 −1.5000 −0.5000)	=	−0.708333	−0.012429
<ss>	8	(−2.0000 −1.0000 0.0000)	=	−0.715278	0.011676
